# The functional and structural alterations of the striatum in chronic spontaneous urticaria

**DOI:** 10.1038/s41598-018-19962-2

**Published:** 2018-01-29

**Authors:** Yuming Wang, Ji-Liang Fang, Bingnan Cui, Jiao Liu, Ping Song, Courtney Lang, Yan Bao, Ruirui Sun, Chenchen Xu, Xu Ding, Zhifang Yan, Yuhe Yan, Qian Kong, Jian Kong

**Affiliations:** 1 Department of Dermatology, Guang’anmen Hospital, China Academy of Chinese Medical Sciences, Beijing, 100053 China; 2 Department of Psychiatry, Massachusetts General Hospital/Harvard Medical School, Charlestown, MA 02129 USA; 3 National-Local Joint Engineering Research Center of Rehabilitation Medicine Technology, Fujian University of Traditional Chinese Medicine, Fuzhou, Fujian, 350122 China; 4 Department of Radiology, Guang’anmen Hospital, China Academy of Chinese Medical Sciences, Beijing, 100053 China

## Abstract

The brain has long been known to be the regulation center of itch, but the neuropathology of chronic itch, such as chronic spontaneous urticaria (CSU), remains unclear. Thus, we aimed to explore the brain areas involved in the pathophysiology of CSU in hopes that our results may provide valuable insights into the treatment of chronic itch conditions. 40 CSU patients and 40 healthy controls (HCs) were recruited. Urticaria activity scores 7 (UAS7) were collected to evaluate patient’s clinical symptoms. Amplitude of low frequency fluctuations (ALFF), voxel-based morphometry (VBM), and seed-based resting-state functional connectivity (rs-FC) analysis were used to assess brain activity and related plasticity. Compared with HCs, CSU patients exhibited 1) higher ALFF values in the right ventral striatum / putamen, which were positively associated with clinical symptoms as measured by UAS7; 2) gray matter volume (GMV) increase in the right ventral striatum and putamen; and 3) decreased rs-FC between the right ventral striatum and the right occipital cortex and between the right putamen and the left precentral gyrus. Using multiple-modality brain imaging tools, we demonstrated the dysfunction of the striatum in CSU. Our results may provide valuable insights into the neuropathology and development of chronic itch.

## Introduction

Chronic spontaneous urticaria (CSU) is a common disorder characterized by the spontaneous eruption of short-lived (<24 h) itchy wheals, with or without angioedema, for a period longer than 6 weeks^1^. CSU affects 0.5–1.0% of the population at any given time and severely diminishes the quality of life of patients^2,3^. Despite its prevalence, there is a lack of understanding of the pathology of CSU and consequently, limited treatment options. Standard therapy with regular doses of non-sedating second-generation H1 antihistamines (H1AH) are ineffective for more than 50% of patients with CSU^2^.

Accumulating evidence suggests that the skin and brain are functionally connected^4,5^. For instance, the brain shares numerous mediators with skin through the hypothalamic-pituitary-adrenal axis (HPA axis)^4^. Studies have shown that the HPA axis may be altered in stress-related skin diseases, resulting in the activation of mast cells^6,7^, which are the primary effector cells in CSU^8^.

The most common clinical manifestation of CSU is repeated itching and scratching. Studies suggest that the brain plays a key role in the itch-scratch cycle^9^. Functional brain imaging studies have identified brain regions associated with the itch-scratch cycle, such as the primary somatosensory cortex (SI), secondary somatosensory cortex (SII), primary motor cortex (MI), premotor cortex (PM), supplementary motor area (SMA), cerebellum, the prefrontal cortex (PFC), the striatum, and thalamus^10–14^.

In recent years, resting-state functional magnetic resonance imaging (rs-fMRI) has been applied to investigate the intrinsic functional organization of the brain^15–17^. There are many resting-state fMRI data analysis methods, some of which focus on local connectivity and examine the properties of spontaneous local brain activity, such as the amplitude of low-frequency fluctuations (ALFF), and others which focus on long-range connectivity among different brain regions, such as seed-based resting state functional connectivity^18^. As a reliable and reproducible data-driven method, ALFF can measure the total power of a given time course within a typical frequency range (e.g., 0.01–0.08 Hz), which has been proven to be a valuable index of regional spontaneous neuronal activity^19,20^. Disruptions in ALFF have been observed in several disorders such as chronic pain^21–24^. In addition to brain function changes, previous studies have also found brain structure changes in patients with chronic itch. For instance, Papoiu *et al*. found gray matter density was significantly increased in the brain stem, hippocampus, amygdala, ventral striatum, and putamen in chronic itch patients with end-stage renal disease^25^.

In the present study, we combined local and long-range resting-state methods with voxel-based morphometry (VBM) analysis to explore the differences in resting state brain activity and brain structure between CSU patients and matched healthy controls (HCs). Many CSU patients suffer from itchy wheals, which generally occur at a specific and consistent time every day, usually during the evening or early morning^26^. To avoid itching intensity variation as a confound, all scans were applied while the CSU patients did not have any itching sensations. We hypothesized that CSU patients would have disrupted brain activity and related plasticity of the brain reward pathways, especially in the striatum.

## Results

### Demographic characteristics

Forty CSU patients (32 female and 8 male) completed the fMRI scan. Forty healthy individuals were matched by age and gender (Table 1).

**Table 1:** Demographic and clinical characteristics between HCs and CSU group. Values are presented as mean ± SD.

	HC (n = 40)	CSU group (n = 40)
Gender (male/female)	8/32	8/32
Age (year)	42.6 ± 10.8	42.6 ± 10.8
UAS7	—	30.8 ± 6.2

### ALFF results

**Figure 1:**
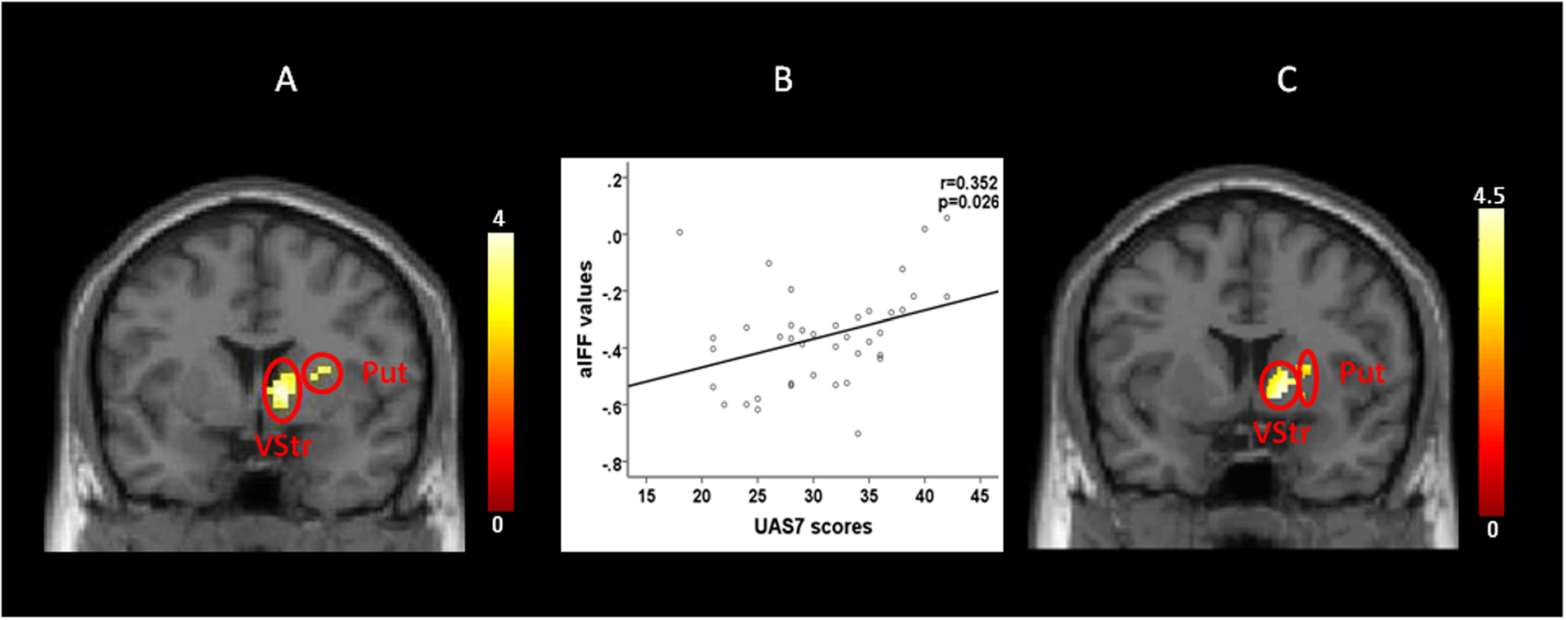
(**A**) Significant differences of ALFF values in right ventral striatum/putamen between the CSU and HCs (CSU > HCs). (**B**) The ALFF values of this cluster in the CSU group were positively correlated with the UAS7 scores (r = 0.352, p = 0.026). (**C**) Increased gray matter volume in CSU compared with HCs in right ventral striatum (VStr) and putamen (Put).

Compared with healthy controls, CSU patients showed significant ALFF increases at the right striatum, including the ventral striatum and putamen (Fig. 1A; Table 2). No other significant regions were identified.

**Table 2:** Brain regions with significant differences in ALFF values among CSU and HCs.

contrast	Brain Regions	MINI coordinates			Peak Z-value	Number of Voxels in the cluster
		X	Y	Z		
CSU > HC	right ventral striatum	12	3	0	3.88	130
	right putamen	24	12	−6	3.46	
HC > CSU	None-					

To further test the association between the ALFF values of the observed cluster and UAS7, we extracted the ALFF values (n = 40) from the right striatum cluster and performed a correlation analysis for CSU patients. The results showed that the ALFF values were positively associated with UAS7 (r = 0.352, p = 0.026) (Fig. 1B).

### VBM results

Significant gray matter volume (GMV) increases were found in the CSU group compared to the healthy control group in the right putamen (cluster size 90, MNI peak coordinates: 30; 14; 6) and right ventral striatum (cluster size 222, MNI peak coordinates: 14; 6; −4) (Fig. 1C).

### rs-FC results

To further investigate the network associated with the striatum, we used the right ventral striatum and putamen, which were identified by VBM analysis, as ROIs and performed a seed-based resting state functional connectivity (rs-FC) analysis.

We found that CSU patients demonstrated decreased rs-FC between the right ventral striatum and right occipital cortex and between the right putamen and left precentral gyrus (Table 3). There were no significant increases observed at the threshold we set.

**Table 3:** Brain regions showing significant functional connectivity differences with the seed.

seed	contrast	Brain regions	Cluster size (voxels)	Peak z-score	MNI coordinates		
					X	Y	Z
right ventral striatum	HCs > CSU	Right occipital cortex	463	4.28	40	−68	20
	CSU > HCs	None-	—	—	—	—	—
right putamen	HCs > CSU	Left precentral gyrus	270	3.97	−36	−12	66
	CSU > HCs	None-					

## Discussion

In this study, we investigated ALFF differences, gray matter volume, and seed based rs-FC between CSU patients and healthy controls. We found that CSU patients exhibited higher ALFF values in the right ventral striatum and putamen. These ALFF values were positively associated with clinical symptoms as measured by UAS7. In addition, significant gray matter volume (GMV) increases were found in the CSU group compared to the healthy group in the right putamen and ventral striatum. Furthermore, seed-based rs-FC analysis showed decreased rs-FC between the right ventral striatum and right occipital cortex and between the right putamen and left precentral gyrus. These findings may provide valuable insights into the neuropathology of chronic itch conditions.

The striatum is one of the nuclei in the subcortical basal ganglia and receives glutamatergic and dopaminergic inputs from different brain regions, as well as serving as the primary input to the rest of the basal ganglia nuclei^27^. It is composed of three nuclei: caudate, putamen, and ventral striatum, and each is associated with different functions, including reward, motivation, reinforcement and motor- and action-planning^28^.

In this study, we found increased ALFF and gray matter volume at the ventral striatum in CSU patients compared with HCs. The ventral striatum plays an important role in processing rewarding stimuli, reinforcing stimuli (e.g., food and water), and stimuli which are both rewarding and reinforcing (addictive drugs, sex, exercise and placebo response)^29,30^. Previous studies suggest that the ventral striatum is involved in the relief of itch by scratching^31^ and is selectively activated during emotional stimulation^32,33^. The most common clinical manifestation of CSU is repeated itchy wheals and scratching. Scratching an itch, a pleasurable experience, is correlated with the intensity of the itch^34,35^. This pleasurable experience is associated with reward regions^36,37^, which are activated by itch even when there is no scratching behavior^38,39^. Molecular mapping also showed an increased number of c-Fos neurons in the ventral striatum and putamen of mice that displayed contagious scratching^40^.

The putamen is a key region within the basal ganglia-thalamic circuit and is involved in action motivation and initiation, as well as in habitual and repetitive behaviors^41,42^. Previous studies have found that the putamen is closely related to the processing of the itch-scratch cycle. Significant activation of the putamen was found during the scratch process of patients with chronic itch^43^ and when an itch was produced by mediators (e.g. cowhage)^44^.

Interestingly, the activity of the putamen during an itch before scratching suggests that this brain region may also code the urge to scratch^43,45^. The putamen can be activated by sedating antihistamines, which may relieve itch by instigating patterns of brain activity similar to those induced by scratching an itch^46^. Thus, the ventral striatum and putamen seems to play an important role in the suppression of itch by the regulation of scratch.

We also found increased gray matter volume at the right ventral striatum and putamen in CSU patients compared with HCs. Although there have been very few studies on brain structure changes in chronic itch patients, an increase of gray matter density and volume in the striatum has been reported in chronic pain^47–50^. The ventral striatum and putamen may be involved in the anticipation of itch, as well as in itch relief. The observed structural changes may be due to the altered function of the ventral striatum and putamen in CSU and compensatory responses to itch and to altered movement control.

To further explore the network involved in the right striatum, we used the ventral striatum and putamen as seeds and performed a resting state functional connectivity analysis. We found that CSU patients were associated with decreased rs-FC relative to HCs between the right putamen and left precentral gyrus. The precentral gyrus is the site of the primary motor cortex, which is the primary region of the motor system and works in association with other brain areas to plan and execute scratching movements^51^. Studies suggest that the primary motor cortex is related to itch processing^10–14^. The primary motor cortex and reward system are associated with motor control and motivational aspects of behavior in the itch-scratch cycle^52^. Scratching in chronic itch patients induces a more robust activation of motor-related regions and of the reward system^52^. The primary motor cortex projects to a portion of the striatum (mostly putamen), which in turn projects back to motor areas of the cortex by way of the ventral anterior and ventral lateral (VA/VL) nuclei of the thalamus^53,54^. We thus speculate that the decreased connectivity between the putamen and primary motor cortex may reflect a disrupted motivation-motor process in chronic itch patients.

We also found that CSU is associated with decreased rs-FC relative to HCs between the right ventral striatum and right occipital cortex. The occipital cortex contains most of the anatomical regions of the visual cortex^55^. A study of mice found that scratching behavior can be induced not only by mere observation of conspecific scratching in a video, but also by artificially stimulating the suprachiasmatic nucleus GRPR neurons, which suggests that visual-related areas may constitute the neural circuits for itch^40^. Further study is needed to explore the role of occipital cortex in chronic itch.

## Conclusions

Using multiple-modality brain imaging tools, we found functional, structural and resting state functional connectivity alterations of the right ventral striatum and putamen in patients with CSU. Our results suggest that CSU may be associated with disrupted reward, motivation, and motor processing.

## Methods

This research protocol has been approved by the Institutional Ethics Committee of Guang’anmen Hospital affiliated with the China Academy of Chinese Medical Sciences. The experiment was performed in accordance with approved guidelines. All subjects signed the written informed consent before the study began.

### Subjects

#### CSU Patients

Forty patients were recruited from the outpatient clinic in the department of dermatology at Guang’anmen Hospital. Patients with a documented history of CSU, characterized by transient, itchy wheals of unknown etiology, occurring regularly from 6 weeks or more before consent acquisition were enrolled in the study. All patients received non-sedation H1-antihistamines (such as Loratadine, Desloratadine, Fexofenadine, Cetirizine dihydrochloride) before enrollment.

Inclusion criteria were: 18 to 60 years old; a UAS7^56^ >14; right handed; have a clear asymptomatic stage more than three hours during the day without non-sedating H1-antihistamine treatment.

Exclusion criteria were: use of sedating antihistamines (such as Alimemazine, Chlorphenamine, Clemastine, Cyproheptadine, Hydroxyzine, Ketotifen, Promethazine), corticosteroids, biologics, psychotropic drugs, or opioids in the past 3 months; chronic pain; pregnant or lactating women; current or history of psychiatric or neurological diseases, head trauma, or loss of consciousness; claustrophobia; metal implants; other skin diseases.

#### Healthy controls (HCs)

Forty right-handed HCs were gender and age matched with CSU patients.

#### Clinical Outcome assessment

Clinical symptoms were assessed using the UAS7^57^ and acquired about three days before the fMRI scan.

#### Magnetic resonance imaging data acquisition

Patients who were taking non-sedating antihistamines discontinued them three days prior to the scan. During the scan, patients did not have any itching or wheals.

Functional MRI was conducted on a 3.0 T Siemens MAGNETOM Skyra MRI system equipped with a standard twenty-channel head coil. Foam pads were used to restrict head motion. T2WI data was acquired to exclude the lesion and abnormality. T1-weighted high resolution structural images were acquired with the three dimensional fast spoiled gradient-echo sequence (TR 5000 ms, TE 2.98 ms, matrix 256 × 256, FOV 256 × 240 mm, FA 1 = 4°, slice thickness 1 mm, gap 0 mm, 176 slices). BOLD fMRI images encompassing the whole brain were collected with the gradient echo EPI sequence (TR 2500 ms, TE 30 ms, matrix 70 × 70, FOV 210 × 210 mm, FA = 90°, slice thickness 3 mm, gap 0 mm, 43 slices, paralleled by AC-PC line). During the 369 second (dummy scan for the first 9 seconds) resting state fMRI scan, subjects were asked to lie still with their eyes closed.

### Statistical analysis

#### Behavioral data

Statistical analyses were performed using SPSS 18.0 (SPSS Inc, Chicago, IL). The mean ± SD were calculated for normally distributed continuous variables.

#### Data preprocessing and Calculation of ALFF

Data preprocessing and calculation of ALFF was performed using DPARSF software of Dpabi V2.3 (a toolbox for Data Processing and Analysis of brain imaging; http://rfmri.org/dpabi)^58^, which is based on Statistical Parametric Mapping 12(SPM12) and the Resting-state fMRI Data Analysis Toolkit (http://www.restfmri.net)^59^ in MATLAB (MathWorks, Natick, MA, USA).

We first checked the scanning image quality for each participant and transformed EPI DICOM to NIFTI. We removed the first 4 time points and then functional slice-timing corrected, spatially realigned, segmented the structural image into gray matter, white matter and cerebrospinal fluid (CSF), removed the friston 24 head motion parameters and CSF signals as regressors, and normalized the images using standard Montreal Neurological Institute (MNI) templates with a resolution of 3 × 3 × 3 mm. Subjects with head movements exceeding 2 mm on any axis or with head rotation greater than 2° were excluded and smoothed with a Gaussian kernel of 8-mm full-width at half maximum (FWHM). Lastly, the time series for each voxel was filtered (bandpass, 0.01–0.08 Hz) to remove the effects of very-low-frequency drift and high frequency noise.

ALFF calculations were based on previous studies^60^. For a given voxel, a fast Fourier transform (FFT) (parameters: taper percent = 0, FFT length = shortest) was used to convert the filtered time series to a frequency domain to obtain the power spectrum. The power spectrum was then square-rooted and averaged across 0.01–0.08 Hz at each voxel, which was deemed as the ALFF. Ultimately, we obtained each participant’s *Z*-standardized ALFF map for the following statistical analysis.

SPM12 was used to examine group differences. Two-sample t-tests were performed between HCs and CSU patients. A threshold of voxel-wise p < 0.005 uncorrected and p < 0.05 cluster-level family-wise error rate (FWE) corrected was applied for ALFF analysis.

#### Voxel-based morphometry (VBM) analysis

VBM analyses were carried out in SPM12. Similar to our previous studies^61^, the T1 images were first segmented into grey matter (GM), white matter and cerebrospinal fluid (CSF) and normalized using the high dimensional DARTEL algorithm^62^. To reduce variability between the subjects, we created a group specific template. Then, the template was used to normalize the images into the standard Montreal Neurological Institute (MNI) space with an isotropic Gaussian kernel of 8 mm full-width at half maximum. Group analysis was applied using two sample t-tests. An absolute threshold of 0.1 was used for masking^61^^,^^63^. Total intracranial volume was obtained by summing up the overall volumes of GM, white matter, and CSF.

In this study, we only focused on the VBM changes in the right ventral striatum / putamen (region of interest) as revealed by ALFF analysis. Small volume correction with a threshold of voxel-wise p < 0.005 (uncorrected) and p < 0.05 FWE corrected at cluster level was applied.

#### Data preprocessing and Calculation of Seed-based Function connectivity

Data preprocessing and calculations of functional connectivity were all carried out using the CONN-fMRI Functional Connectivity toolbox v17.c^64^ (http://www.nitrc.org/projects/conn), which is based on SPM12 in MATLAB. We used the default preprocessing pipeline for volume-based analysis (direct normalization to MNI-space). The specific steps are as follows: functional realignment and unwarped; functionally centered to coordinates; functional slice-timing corrected; structural center to coordinates; structural segmentation and normalization; functional normalization; functional outlier detection (ART-based identification of outlier scans for scrubbing); functional smoothing with an 8-mm FWHM. Band-pass filtering was performed using the low frequency band (0.01–0.08 Hz).

Seeds were chosen based on the VBM results (right ventral striatum and right putamen), which indicate the overlap area in both ALFF and VBM analysis. Functional connectivity measures were computed between each seed and every other voxel in the brain. The residual BOLD time course was extracted from a given seed and then its first-level correlation map was estimated by computing Pearson’s correlation coefficients between that time course and the time courses of all other voxels in the brain. Correlation coefficients were transformed into Fisher’s ‘Z’-scores to increase normality and allow for improved second-level General Linear Model analyses. Seed-to-voxel functional connectivity was estimated for each subject.

SPM12 was used to examine group differences. Two-sample t-tests were performed between HCs and CSU. A threshold of voxel-wise p < 0.005 uncorrected and cluster-level p ≤ 0.05 FWE corrected was applied for rs-FC data analysis.
